# Resolvin D1 improves allograft osteointegration and directly enhances osteoblasts differentiation

**DOI:** 10.3389/fimmu.2023.1086930

**Published:** 2023-02-27

**Authors:** Noy Pinto, Yehuda Klein, Eilon David, David Polak, Daniel Steinberg, Gilad Mizrahi, Yasmin Khoury, Yechezkel Barenholz, Stella Chaushu

**Affiliations:** ^1^ Institute of Dental Sciences, Faculty of Dental Medicine, Hebrew University of Jerusalem, Jerusalem, Israel; ^2^ Institute for Medical Research Israel-Canada, Faculty of Medicine, Hebrew University of Jerusalem, Jerusalem, Israel; ^3^ Department of Orthodontics, Hadassah Medical Center, Faculty of Dental Medicine, Hebrew University of Jerusalem, Jerusalem, Israel; ^4^ Department of Periodontics, Faculty of Dental Medicine, Hebrew University of Jerusalem, Jerusalem, Israel; ^5^ The Lautenberg Center for Immunology and Cancer Research, Department of Immunology and Cancer Research-Medical Research, Israel-Canada (IMRIC), Jerusalem, Israel; ^6^ Department of Developmental Biology and Cancer Research, Institute for Medical Research Israel-Canada, Faculty of Medicine, Hebrew University of Jerusalem, Jerusalem, Israel

**Keywords:** bone, resolvin D1, allograft, regeneration, osteoblasts

## Abstract

**Introduction:**

Allografts are the most common bone grafts for repairing osseous defects. However, their use is associated with an increased risk for infections, donor disease transmission and osteointegration deficiency. Resolvin D1 (RvD1) is an endogenous lipid with a scientifically proven pivotal role in inflammation resolution and osteoclastogenesis inhibition. Yet, its biological relevance as a potential bone regenerative drug has been scarcely studied. Here, we aim to investigate the RvD1 effect on allograft osteointegration in the alveolar bone regeneration (ABR) murine model.

**Methods:**

ABR model consisted of osseous defects that were generated by the extraction of the maxillary first molar in C57BL/6 mice. The sockets were filled with allograft and analyzed *via* RNA sequencing. Then they were locally injected with either RvD1 or saline *via* single or repeated administrations. The mice were sacrificed 2W after the procedure, and regenerated sites were analyzed using *µ*CT and histology. First, MC3T3-E1 preosteoblasts were plated with IL-17 pro-inflammatory medium, and RANKL/OPG ratio was measured. Secondly, the MC3T3-E1 were cultured w/o RvD1, for 3W. Osteoblasts’ markers were evaluated in different days, using qRT-PCR and Alizarin Red staining for calcified matrix.

**Results:**

*In vivo*, neither allograft alone nor single RvD1 administration promote bone regeneration in comparison to the control of spontaneous healing and even triggered an elevation in NR1D1 and IL1RL1 expression, markers associated with inflammation and inhibition of bone cell differentiation. However, repeated RvD1 treatment increased bone content by 135.92% ± 45.98% compared to its specific control, repeated sham, and by 39.12% ± 26.3% when compared to the spontaneous healing control group (n=7/group). Histologically, repeated RvD1 reduced the number of TRAP-positive cells, and enhanced allograft osteointegration with new bone formation. *In vitro*, RvD1 rescued OPG expression and decreased RANKL/OPG ratio in IL-17 pro-inflammatory conditions. Furthermore, RvD1 increased the expression of RUNX2, OSX, BSP and OC/BGLAP2 and the mineralized extracellular matrix during MC3T3-E1 osteoblasts differentiation.

**Conclusions:**

Repeated administrations of RvD1 promote bone regeneration *via* a dual mechanism: directly, *via* enhancement of osteoblasts’ differentiation and indirectly, through reduction of osteoclastogenesis and RANKL/OPG ratio. This suggests that RvD1 may be a potential therapeutic bioagent for osseous regeneration following allograft implantation.

## Introduction

Despite the gradual growth in the graft industry, there is still a growing demand for innovative treatments for bone deficiencies in orthopedics, implantology and general dentistry (1). Evidence suggests that treatments with bone substitutes alone may result in insufficient bone repair, and optimal bone healing is dependent on two major aspects: an active stimulating of bone growth and bone remodeling. For stimulating bone growth, a combination of local presence of bioactive bone growth stimulators with grafts is considered a good strategy to improve bone regeneration (2–4). Some familiar bone growth stimulators are Bone Morphogenic Proteins (BMPs), which are released in osteoclastic resorption and promote osteoblasts differentiation in bone healing process (5). Other biomaterials, such as Vascular Endothelial Growth Factor (VEGF), Transforming Growth Factor beta (TGF-β), Platelet Derived Growth Factor (PDGF), stimulate migration and differentiation of osteoprogenitor cells to the injury site. VEGF enhances blood vessels growth and formation of callus in bone fracture healing, while TGF-β mostly promotes osteoblasts chemotaxis (2), and therefore, promote bone healing. However, there are still concerns related to these stimulators. Their efficiency in humans is controversial (6), there is a lack of data on the optimal therapeutic dose and some of them, such as BMPs, have been associated with potential carcinogenic side effects (7, 8).

Bone remodeling is based on the complex interplay between the skeleton and immune system, an interplay termed recently ‘Osteoimmunology’. Optimal bone recovery is mediated by the recruitment of immune cells to the site, and secretion of multiple factors. In the inflammatory phase of fracture healing, platelets, neutrophils and macrophages invade to the injury site, and secrete growth factors and cytokines that promote mesenchymal cells to arrive (9). However, these inflammatory signals are limited and temporary. Therefore, the resolution of inflammation is also beneficial for optimal bone remodeling and healing. It is an active process that is mediated in part by specialized pro-resolving lipid mediators (SPMs), such as Resolvins, Protectins and other derivates of omega-3 fatty acids (10).

Nowadays, there is no drug which can promote bone healing or graft osteointegration *via* stimulation of bone deposition and controlling the inflammation.

Resolvin D1 (RvD1) (7S,8R,17S-trihydroxy-4Z,9E,11E,13Z,15E,19Z-docosahexaenoic acid), a derivative of docosahexaenoic acid (DHA), is efficient in treating inflammation across a wide variety of inflammatory conditions such as bowel disease, acute lung injury (covid-19), peritonitis and heart failure (11). In addition to its pro-resolving activity in an inflammatory environment, RvD1 has an anti-catabolic effect in diseases that are accompanied by bone loss and tissue degradation. RvD1 abolishes a few factors which are involved in osteoarthritis in human chondrocytes (12), thus has the potential to serve as a target for other rheumatic diseases (13). RvD1 contributed to joint protection in the murine arthritis model by inhibition of cartilage resorption (14). RvD1 also decreased osteoclast differentiation and activation *in vitro* and decreased bone and joint destruction *in vivo* (15). We also previously showed that RvD1 affected immune cells expression and decreased osteoclastogenesis in orthodontic tooth movement (16).

In addition to its anti-catabolic effect, some evidence implies that RvD1 is capable of actively preserving bone. RvD1 embedded in chitosan scaffolds improved bone healing (17). However, there is no mechanism showing the direct effect of RvD1 on bone deposition. In a recent *in vitro* study, IL-6 was introduced with its receptor in osteoclast cultures and decreased RANKL and OPG expression, while stimulation of these cells with RvE1 increased OPG without any change in RANKL expression (18). Another *in vitro* experiment included neonatal calvaria cells that were treated with testosterone which decreased the expression of OC, OPG and RANKL. RvD2 treatment restored their expression levels to baseline (19).

Accordingly, we here hypothesize that RvD1 might have an anabolic effect and increase bone formation by actively promoting osteoblasts functionality. Subsequently, we aim to assess the potential therapeutic activity for RvD1 as a bone healing stimulator when combined with allograft in tooth extraction sockets. Tooth extraction might result in complications such as ridge bone loss (20), socket infection (21) and poor repair in some alveolar sockets that are not treated with bone grafts (22). In addition, some cases require alveolar bone augmentation before inserting dental implants due to insufficient bone mass and proximity to limiting anatomical structures.

Our results provide promising evidence for a novel anabolic effect of RvD1 in bone remodeling. Although we focused on tooth socket healing, the results might be relevant in several other fields, such as non-union fractures and degenerative diseases.

## Materials and methods

### Animals

The study was approved by the Animal Care and Use Committee of the Hebrew University. 8 weeks old C57BL male mice at weight of 20 grams were purchased from Harlen, Israel and maintained under specific pathogen-free (SPF) conditions at the Ein-Kerem campus of the Hebrew University, Jerusalem. Mice were kept at 25^0^C with a 12–24 hours light/dark cycle and given free access to food and water.

### Bone allograft preparation

Murine allograft was prepared from femurs and tibias as previously described (23).

Briefly, long bones (femurs and tibias) from C57BL/6 mice, 6 to 8 weeks old, were harvested using fine forceps and a scalpel to remove the surface periosteum and the marrow cavity was flushed with phosphate buffered saline (PBS) to remove free bone marrow cells. The graft was then washed with hydrogen peroxide for 10 minutes, followed by PBS wash and incubation in tert‐butyl alcohol overnight. Next, grafts were thoroughly rinsed in PBS to remove residual tert‐butyl alcohol, fast freeze at −196°C and lyophilized overnight. The dried bones were thawed to room temperature, pulverized using mortar and pestle to give an average of 65.60 ± 20‐μm particle size. Finally, allograft particles were immersed into PBS in cell culture conditions (37°C, 5% CO2) for 24 hours. Protein quantification of the allograft particles was performed using micro bicinchoninic acid assay to ensure no protein as well as live cell in the prepared allograft bones. Before implantation, bones were ultraviolet irradiated for 30 minutes to ensure sterility. The absence of proteins in the particulate bone material was validated (data not shown).

### Allograft implantation combined with free RvD1 in alveolar bone regeneration (ABR) murine model

ABR model was performed in C57BL mice, as previously described (24, 25). Then, the mice were randomly divided according to the administrated treatment (n=7/group): (a) first treatment group received allograft particles mixed with RvD1 (allograft + single RvD1) (b) second treatment group received allograft particles mixed with RvD1 and 3 additional administrations of RvD1 at 4, 7, 10 days post ABR, (c) first control group in which alveolar bone was allowed to heal spontaneously without allograft (Spon. Healing), (d) second control group received allograft mixed with saline (allograft +single sham), (e) third control group received allograft mixed with saline + 3 gingival injections of saline at 4, 7, 10 days post ABR, to examine the effect of repetitive tissue injury on bone healing (rep-sham). The administration of RvD1 (15 μl, 0.51 μg/ml: 0.76 mg, per administration) or saline was performed as previously described (23).

All mice were sacrificed 2 weeks post ABR, and maxillae were harvested for analysis.

### Sample preparation

For RvD1 Elisa, maxillae were harvested and the alveolar bone socket, PDL, and gingiva were pulverized in a homogenizer and kept in 500 μl of sterile saline histidine (pH=7). All samples were immediately frozen in liquid nitrogen and kept in -80 until the RvD1 Elisa assay and according to manufactures instruction.

For radiographic and histologic analysis, maxillae were harvested and fixed in 4% paraformaldehyde (pH 7.4) in PBS for 1 day at 4°C and then kept in 70% ethanol. Following the scanning, samples were prepared for histological sections and examinations previously described (26).

### Micro-Computed Tomography (μCT) imaging and ABR measurement

Maxillae were scanned *via* μCT40^®^, Scanco Medical, Brüttisellen, Switzerland (24) as previously described. Morphological parameters of trabecular bone microarchitecture were assessed according to guidelines as previously described (27, 28). The borders of the regenerated bone sites were marked according to a cylindrical region of interest (ROI) in all the samples mesial to M2 with an axis depth/length of 350 μm (150‐ to 500‐μm below the M2 root furcation) and a diameter of 700 μm. two‐dimensional microarchitecture measurements were included and calculated: bone volume/total volume (BV/TV, %) and bone mineral density.

### Hematoxylin and eosin, Tartrate- resistant acid phosphatase, and Masson Trichrome staining

Maxillae bones were decalcified in 10% (w/v) ethylenediaminetetraacetic acid (EDTA, pH 7.4) for 10 days. Then, samples were embedded in Optimal Cutting Temperature compound and sagittal slices of 10‐μm‐thick cryo‐sections were performed.

For Massons’s Trichrome staining, samples were stabilized in preheated Bouin’s solution at 56°C for 15 minutes, then washed in tap water and stained in Weigert’s Iron Hematoxylin Solution for 5 minutes (nuclei staining). Stained samples were then washed with Biebrich Scarlet-Acid Fucshin for 5 minutes (cytoplasm & muscle staining). Subsequently, samples were placed with phosphotungstic and phosphomolybdic acid followed by soaking the samples in Aniline Blue Solution for 5 minutes (collagen fibers staining) were then rinsed with Acetic Acid, 1%, for 2 minutes followed by rinsing and dehydration with alcohol and xylene. Slides were then mounted for additional analysis (Kit components from Sigma- Aldrich).

For Tartrate‐resistant acid phosphatase staining (TRAP) staining, with hematoxylin counterstaining was performed according to the manufacturer’s instructions and enabled quantification of the osteoclasts (N=3-4/group).

Following H&E, TRAP and Masson-Trichrome staining, specimens were examined and photographed with high-quality microscope (Nikon eclipse 90i, Tokyo, Japan). Images captured using x2 and x10 magnification. Morphometric analysis included visual observation of osteoid and allograft particles. To assess *de-novo* osteoid apposition, a grid sized 640X488 µm of the regenerated site (the area of M1 socket) was analyzed by ImageJ software (N=3-4/group).

### RNA extraction for mRNA sequencing, qRT-PCR and quality control

Maxillae were pulverized in a homogenizer, and total RNA was isolated from bones lysates or from cultured cells with TriZol (1000 ul per sample; Thermo Fisher Scientific). RNA purity was detected with a Nanodrop. DNA-free RNA was obtained by using and RNeasy Mini Kit (QIAGEN) with DNase treatment according to the manufacturer’s instructions. For quality control of RNA extraction yield, an RNA Screen Tape kit (Agilent Technologies), a D1000 Screen Tape kit (Agilent Technologies), Qubit RNA HS Assay kit (Invitrogen) and a Qubit DNA HS Assay kit (Invitrogen) were used for each specific step. RNA samples from Allograft vs Spon. Healing groups were all passed quality control analysis on a Bioanalyzer 2100 (Agilent Technologies). The 3 biological replicates with the highest RNA were used for mRNA library preparation and bioinformatics analysis as described previously (29).

### mRNA library preparation

RNA concentration was measured using Qubit 4 Fluorometer (Thermo Fisher Scientific) and RNA quality was measured using Agilent 2200 Tape Station (Agilent). RNA sequencing libraries were prepared using the CEL-Seq2 protocol, as published by (30) with minor modifications. Instead of single cells as input, 2 ng purified RNA was taken as input for library preparation. The CEL-Seq2 libraries were sequenced on an Illumina NextSeq 550 sequencer (Illumina). RNA measurements, library preparation and sequencing were performed by the Technion Genome Center, Technion, Israel.

### Trimming and filtering of raw reads

Quality trimming was done at the 3’ end using cutadapt. The quality cutoff was 10, skipping all G bases (that can indicate a lack of signal in Next-Seq’s two-color chemistry). The parameter was –nextseq-trim=10. Also using cutadapt, adapter and poly-A sequences were removed. The error rate (-e parameter) was set to zero. Reads that became shorter than 28 nt were filtered out (-m parameter).

### Alignment and counting

The processed fastq files were aligned to the Mus musculus transcriptome and genome using TopHat. The genome version was GRCm38 with annotations from Ensembl release 99. ERCC spike-in sequences (positive controls of CEL-Seq protocol) were aligned as well to the DNA Sequence Library SRM-2374. Strand information was taken into consideration (–library-type fr-secondstrand). Alignment allowed up to 2 mismatches per read, and a total edit distance of 5.

Quantification was done using htseq-count. Reads that aligned with a quality lower than 10 were skipped. Strand information was taken into consideration (–stranded=‘yes’). An annotation file that lacked information for genes of type IG, TR, Artifact, miRNA, Mt_rRNA, Mt_tRNA, ncRNA, piRNA, pre_miRNA, rRNA, ribozyme, sRNA, scRNA, scaRNA, siRNA, snRNA, snoRNA, tRNA and vaultRNA was used.

### Differential expression

Differential expression analysis was done with the DESeq2 package. Genes with a mean of counts less than 3 over all samples were filtered out, then size factors and dispersion were calculated. Normalized counts were used for several quality control assays, such as distance heatmaps and principal component analysis, which were calculated and visualized in R. Differential expression was calculated with default parameters except not using the independent Filtering algorithm. Significance threshold was taken as padj<0.1 (default). Finally, the results were combined with gene details (such as symbol, known transcripts, etc.), taken from the results of a BioMart query (Ensembl, release 99), to produce the final Excel file.

### qRT-PCR

mRNA was reverse-transcribed into cDNA using a High-Capacity cDNA Reverse Transcription Kit (Tamar laboratory supplies Ltd). The resultant cDNA was subjected to qRT-PCR using the qPCRBIO SyGreen Blue Mix Hi-Rox (Tamar laboratory supplies Ltd) The mRNA expression was normalized to murine GAPDH1 (Glyceraldehyde-3-phosphate dehydrogenase). Murine primers sequences used for reverse transcription- quantitative PCR. **Supplementary Data Table S1**.

### MC3T3-E1 cell culture

The MC3T3-E1, Subclone- 4 were purchased from ATCC and used as osteoblastic primary cell line. MC3T3-E1 cells were cultured in Mem- Alpha medium w/o ascorbic acid (Rhenium) containing 10% FBS, 1% streptomycin and 1% glutamine. Cells were cultured at 37°C in a humidified 5% CO2 atmosphere. MC3T3-E1 cells were cultured in a 6 well plates at a density of 10^4^ cells per 1 ml (total 2 ml per well).

### RvD1 effect on RANKL/OPG in inflammatory condition *in vitro*

Cells were cultured with IL-17 (50 nM,Pepro Tech) and with or without RvD1 (200 nM) for 24 hours. Cells without IL17 nor RvD1 treatment served as a control. RNA was extracted and RANKL and OPG expression were measured.

### RvD1 effect on osteoblastogenesis *in vitro*

To promote osteoblasts differentiation, cells were cultured with a supplemented medium consisted of ascorbic acid (280 mM) and β-glycerophosphate (10 mM) with or without RvD1 (200 nM). Medium was changed every other day. RNA was extracted from cells in days 5, 7, 9, 14 and 21. The extracellular calcium was analyzed *via* Alizarin Red staining in days 10, 14 and 21.

### Alizarin red staining

The extracellular calcium deposition was stained *via* alizarin red staining protocol (31, 32). The medium was removed, and cells were washed with PBS three times and fixed with 4% paraformaldehyde. Subsequently cells were washed with PBS 3 times and stained in filtered 40 mM alizarin red (pH ~ 4.2) for 15 minutes in room temperature (Alizarin Red powder, Cas Number 130-22-3 Sigma). Finally, cells were washed, and the wells photographed in stereomicroscope (SMZ25) to identify calcified nodules in bright- orange- red color. For numerical quantification (33), stained cells were dissolved in 10% acetic acid. The suspended samples were heated to 80°c for 10 minutes. Then, samples were cooled on ice for 5 minutes, and centrifuged at 20,000 g for 15 minutes. Supernatant pH was adjusted to 4.2 and absorbance was measured in 405 nM.

### Statistical analysis

The gene expression data were analyzed as mentioned above. In other experiments, the data were analyzed with GraphPad Prism v.8 (GraphPad Software, San Diego, CA, USA) and the numerical values obtained are expressed as the means ± SEM. Normally distributed data were analyzed with paired t-test. The asterisk symbol represents a statistical significance *<0.05 **< 0.01 ***< 0.005..

## Results

### Allograft implantation did not improve bone healing vs. spontaneous healing at 2 weeks post ABR

**Figure 1:**
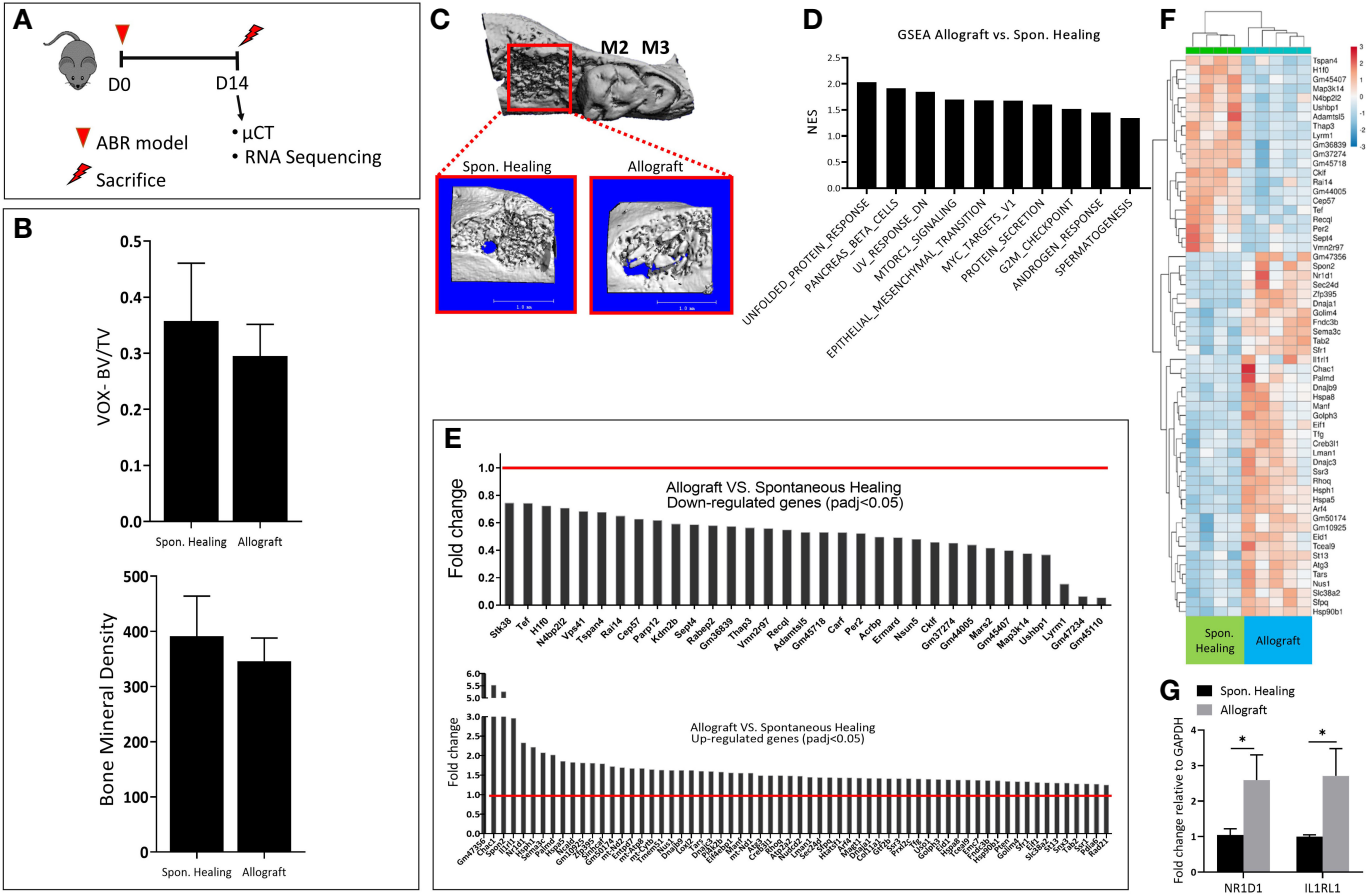
Bone healing two weeks following alveolar bone regeneration with Allograft vs. Spontan. Healing. **(A)** Experimental timeline: 8 weeks old male mice (C57BL =7/group) were divided into two groups; the control group of a tooth extraction without allograft implantation (Spon. Healing), an experimental group with allograft implantation, were sacrificed for radiographic and mRNA sequencing analysis 2 weeks post-procedure. **(B)** Numerical µCT evaluation of alveolar socket: bone volume/total volume (BV/TV) and Bone Mineral Density. **(C)** µCT 3- dimensional analysis of the regenerated alveolar bone (marked in red square), with magnification. **(D)** Upregulated GSEA pathways in allograft treatment. **(E)** The highest differentially down\ up-regulated genes. **(F)** Heatmap showing the highest 60 Differentially expressed genes (DEGs) **(G)** mRNA validation for genes of interest (NR1D1, IL1RL1).

According to the experimental timeline (**Figure 1A**), 2 weeks post ABR radiographic numerical analysis of the socket showed that allograft did not improve the BV/TV ratio and the bone mineral density (**Figures 1B, C**).

For a deeper understanding of the underlying molecular changes induced by allograft osteointegration at this time point, we conducted mRNA sequencing. A few pathways were upregulated in allograft compared to Spon. Healing, including MTORC and androgenic response (**Figure 1D**). The gene set enrichment analysis (GSEA) showed a total of 97 differentially expressed genes (DEGs). Among them, 33 DEGs were downregulated and 64 DEGs were upregulated in allograft vs. Spon. Healing. (**Figure 1E**), These findings were also supported by the heatmap analysis (**Figure 1F**). To validate the sequencing data, genes of interest including NR1D1 and IL1RL1 were assessed by qRT- PCR (**Figure 1G**).

We also monitored the changes in mice weight during the experiments, to investigate their rehabilitation after surgery. Allograft treatment mice displayed lower values of body weight post-surgery, compared to the Spon. Healing group (**Figure S1C**) (n=7/group, *p<*0.005).

### Rep-RvD1 administrations improve bone volume and density

**Figure 2:**
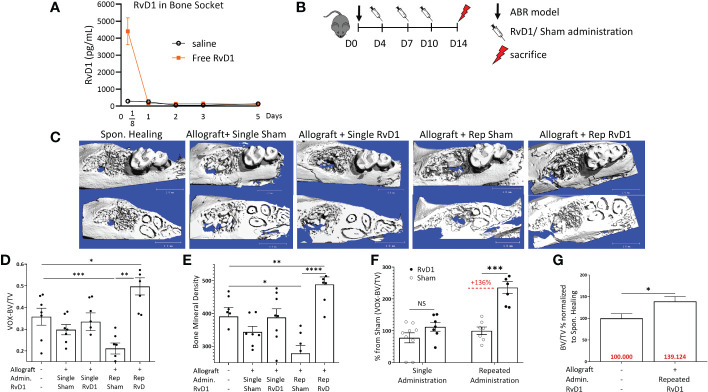
Rep-RvD1 improved bone healing. **(A)** RvD1 concentrations in bone sockets, in different time points (days) post ABR procedure (pg/mL) (n=4-6/group). **(B)** Experimental timeline: 8 weeks old male mice (C57BL n=7/group) were divided into five groups. Control group mice were operated for ABR without allograft implantation (Spon. Healing), an experimental group of mice received allograft combined with RvD1 (60 ug/ml 15 ul) Allograft + single RvD1); the control group of allografts combined with saline (Allograft + single sham). Another experimental group of mice received allograft combined with RvD1 (60 ug/ml 15 ul), in addition to three more intra- palatal RvD1 injections (60 ug/ml 15 ul) in days 4, 7, 10 post-ABR (Allograft + Rep-RvD1), beside the control group which received allograft combined with saline (15 ul), followed with three more intra- palatal saline injections (15 ul) in days 4, 7, 10 post-ABR (Allograft + Rep-sham). All mice were sacrificed in 2 weeks post ABR procedure for radiographic analysis. **(C)** µCT 3- dimensional analysis of the regenerated alveolar bone, the occlusal plane of molars (above images), and the roots plane (below images). **(D)** Bone volume/total volume (BV/TV) numerical evaluation values for all groups. **(E)** Bone mineral density numerical evaluation for all groups. **(F)** BV/TV of RvD1 administration normalized the specific control group (%). **(G)** BV/TV of rep-RvD1 administration combined with allograft normalized to the Spon. Healing control group (%). The asterisk symbol represents a statistical significance *<0.05; **< 0.01; ***< 0.005; **** <0.001 .

Following saline injection in the ABR socket, RvD1 was found in only small amounts (186.82 ± 93.9 pg/ml), indicating no significant endogenous RvD1 production post-surgery or post allograft augmentation. In contrast, the RvD1 treatment sockets exhibited high RvD1 levels (4403.9 ± 1765.4 pg/ml) at 3 hours post administration. However, 1 day post procedure, the RvD1 values dropped significantly to levels similar to the saline groups (146.9 ± 61.5 pg/ml) (**Figure 2A**).

Therefore, to maintain an adequate level of RvD1 at the target site, we repeated its gingival administrations 3 more times during the 2 weeks follow-up period (**Figure 2B**) Rep-RvD1 had a significant impact on bone quality parameters when compared to all allograft control groups, especially when compared to its specific control (rep-sham), as displayed by the µCT 3D analysis (**Figure 2C**). Rep-RvD1 elevated BV/TV compared to rep-sham (0.49 ± 0.09 vox vs. 0.21 ± 0.06, *p*=0.007) and to Spon. Healing (0.49 ± 0.09 vox vs. 0.35 ± 0.09 vox, respectively, *p*=0.013) (**Figure 2D**). Single RvD1 treatment displayed no benefit in bone regeneration compared to single-sham control (BV/TV ratio 0.33 ± 0.1 vox vs. 0.29 ± 0.05, respectively, *p*>0.05) (**Figure 2D**). When rep-sham was considered 100%, rep-RvD1 elevated the BV/TV ratio by 135.92% ± 45.98% (*p*= 0.001) (**Figure 2F**). In contrast, when Spon. Healing was considered 100%, rep-RvD1 elevated the BV/TV ratio by 39.12% ± 26.3% (**Figure 2G**).

Rep-RvD1 also elevated the bone mineral content compared to rep-sham (488.48 ± 56.21 vs. 278 ± 59.3, respectively, *p<*0.001) and to Spon. Healing (488.48 ± 56.21 vs. 391.16 ± 67.4, respectively, *p*=0.009) while single RvD1 had no impact on the bone mineral content compared to single sham (379.79 ± 71.3 vs. 343.58 ± 41.7, respectively) (**Figures 2E, F**).

The experiments presented in **Figure 2** clearly show that prolongation of the exposure of bone to RvD1 during healing, results in superior therapeutic effects. However, repeated drug administration has drawbacks since it requires repeated anesthesia and injures the tissue several times (**Figure 2B**). Therefore, it was not surprising to find that the rep-sham control group was associated with poor BV/TV ratios in comparison to single sham treatment (**Figure S2A**). The same trend was reflected also in the bone mineral content (**Figure 2D**). Furthermore, 1 day after the first intra-palatal injection (day 8 post ABR), a significant weight loss was found in repeated RvD1 & sham administration groups, compared to allograft single treatment groups (**Figure S2B**).

### Rep-RvD1 increases allograft osteointegration and enhances bone remodeling at the cellular and molecular level

**Figure 3:**
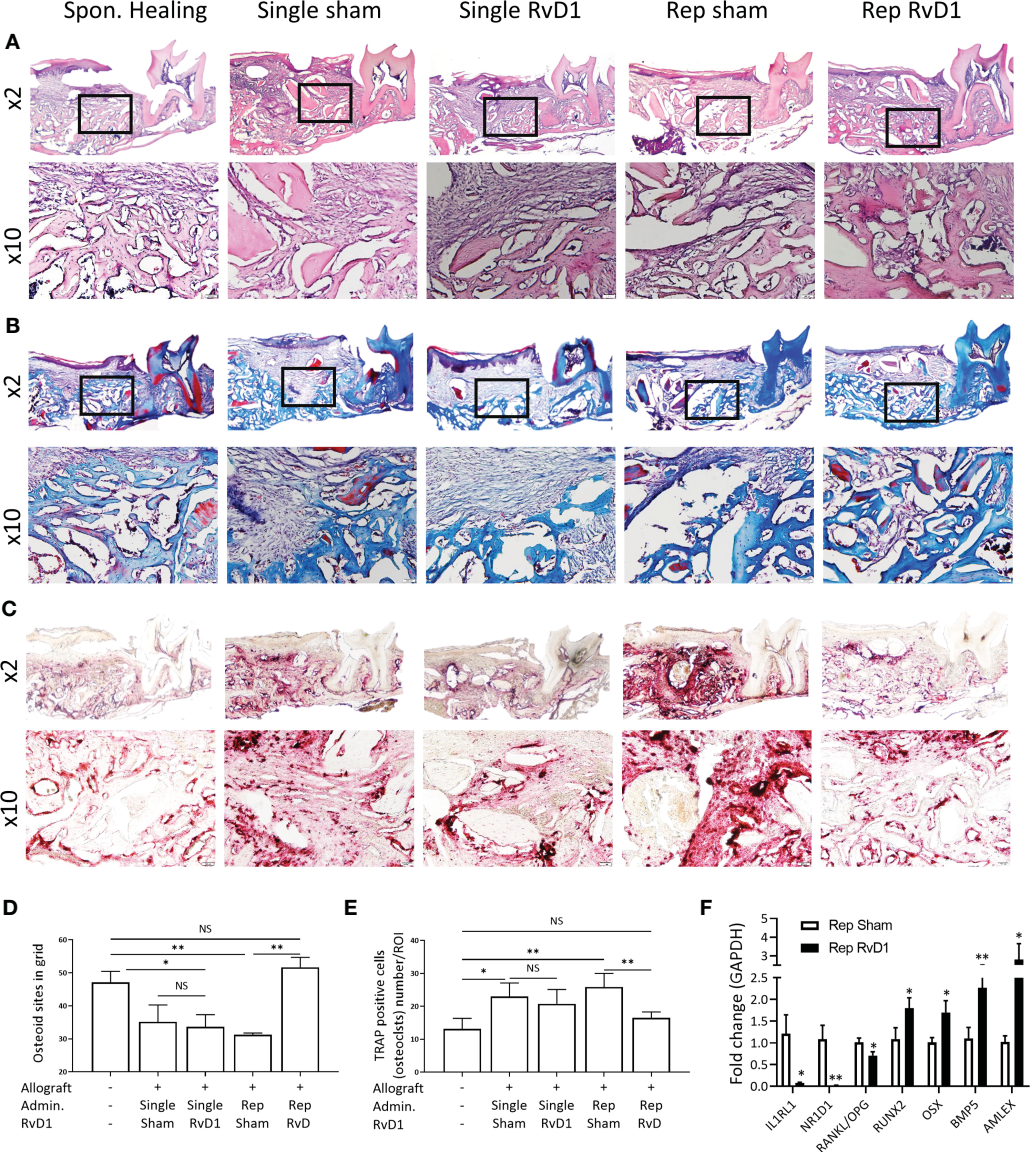
Rep-RvD1 increased osteoid apposition and decreased osteoclasts. Representative histological images are presented at x2 and x10. **(A)** Hematoxylin & Eosin staining. **(B)** Trichrome (Masson) staining. **(C)** TRAP staining. **(D)** Numerical evaluation of *de-novo* osteoid apposition. **(E)** Numerical evaluation of osteoclasts/ROI. **(F)** Fold change of mRNA expression in rep-RvD1 vs. rep-sham bones samples. The asterisk symbol represents a statistical significance *<0.05; **< 0.01.

Following the radiographical analysis, we proceeded with histological evaluation. Allograft particles were observed at two weeks post-implantation, as well as woven bone (**Figure 3A**) Trichrome (Masson) staining distinguished the allograft particles in red and the woven bone in blue. Rep-RvD1 showed higher woven bone content in the alveolar bone defect and improved osteointegration between allograft and new bone (mixed red allograft with new blue bone), in comparison to single allograft treatments with or without RvD1. The rep-sham exhibited sparse collagen fibers, and relatively big allograft particles lacking significant osteointegration with new bone. (**Figure 3B**) Numerical grid evaluation of novel osteoid apposition demonstrate that all allograft treatment groups displayed decreased osteoid apposition compared with Spon. Healing, except rep-RvD1. Rep-RvD1 exhibited statistically significant increased osteoid apposition sites compared with all other groups (**Figure 3D**) This is indicative of enhanced osteoblastic activity *in- vivo*.

The osteoclast’s number (TRAP-positive multinuclear cells) was increased in all allograft groups, except in the rep-RvD1 group, in which it dramatically decreased (**Figures 3C, E**).

Following histological and radiographical analysis, we quantified the mRNA expression of key markers *in vivo* to investigate the RvD1 mechanism of action in ABR.

Rep-RvD1 significantly decreased IL1RL1 0.07 ± 0.03 and NR1D1 0.02 ± 0.006 and the RANKL/OPG ratio (0.7 ± 0.15)), compared with rep-sham treatment group (1.2 ± 0.7 *p*= 0.01, 1.08 ± 0.4 *p*=0.005, 1 ± 0.17, *p*=0.02, respectively) (**Figure 3F**).

In contrast, rep-RvD1 enhanced the expression of key markers of osteoblastic differentiation, survival and activity, such as RUNX2 (RUNX Family Transcription Factor 2) (1.79 ± 0.411) and OSX (Osterix) (1.7 ± 0.46), in comparison to rep-sham (1.08 ± 0.45, *p*=0.04 and 1.01 ± 0.15, *p*=0.03, respectively).

In addition, rep-RvD1 increased the expression of anabolic factors, such as BMP-5 (2.26 ± 0.42) and AMELX (Amelogenin) (2.79 ± 1.48) in comparison to rep-sham (1.1 ± 0.43, *p=*0.008 and 1.02 ± 0.24, *p*=0.04, respectively) (**Figure 3F**).

### RvD1 increases OPG expression and the OPG/RANKL ratio in inflammatory environment *in vitro*

**Figure 4:**
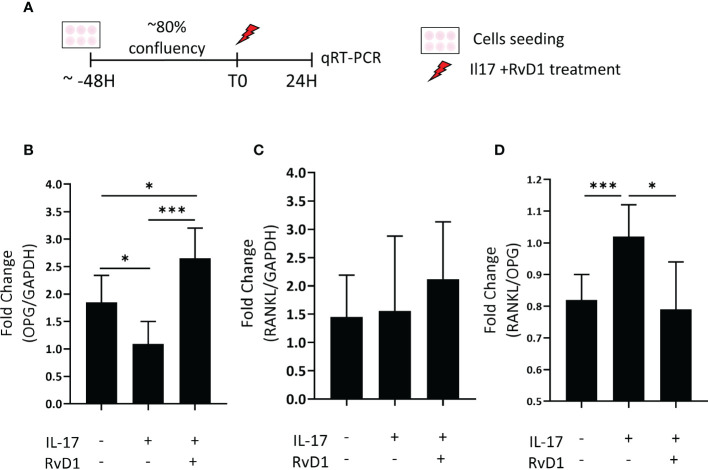
RvD1 increased OPG and decreased the RANKL : OPG ratio expression inflammatory conditions *in vitro.* **(A)** MC3T3E-1 pre-osteoblasts were stimulated with IL-17 (50 nM) with and without RvD1(200 nM) for 24 hours. RNA was extracted from cells followed by RANKL, and OPG mRNA evaluation by qRT-PCR. Unstimulated cells were analyzed as an experimental control group. **(B)** OPG mRNA expression in cells normalized to stimulated cells without RvD1 treatment. **(C)** RANKL mRNA expression in cells normalized to stimulated cells without RvD1 treatment. **(D)** RANKL/OPG ratio mRNA expression in cells normalized to stimulated cells without RvD1 treatment. The asterisk symbol represents a statistical significance *<0.05; ***< 0.005.

To investigate whether RvD1 can modulate osteoblasts in an inflammatory environment (induced by IL-17), the mRNA expression of OPG and RANKL were determined in MC3T3-E1 osteoblasts primary cells line cultured with or without RvD1 (200 nM), for 24 hours (**Figure 4A**). RvD1 rescued the OPG (**Figure 4B**) but did not significantly alter RANKL expression (**Figure 4C**). Consequently, the RANKL/OPG ratio was decreased (**Figure 4D**).

### RvD1 increases osteoblasts’ differentiation and calcium deposition *in vitro*

Next, we proved that RvD1 is capable to enhance the expression of osteoblastic key markers of functionality and differentiation.

**Figure 5:**
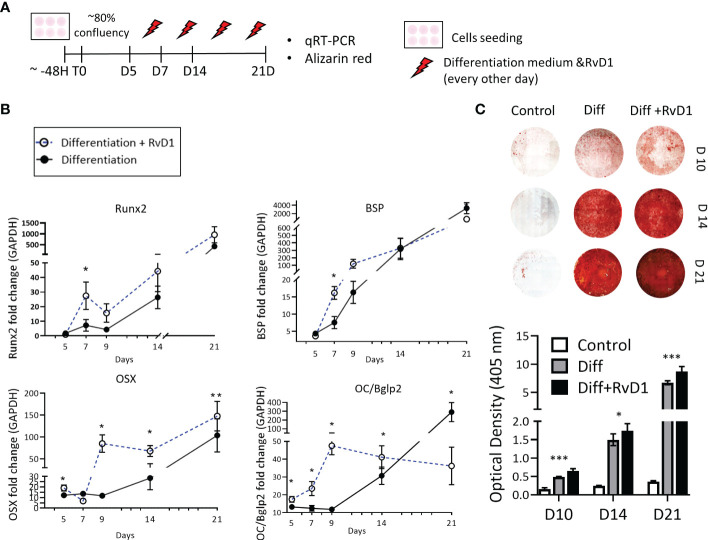
Resolvin D1 increased osteoblasts differentiation and calcium deposition. **(A)** Experimental timeline: MC3T3E-1 pre- osteoblasts cells were plated and treated with differentiation supplemented medium (materials& methods) with and without RvD1 (200 nM) for 21 days. Undifferentiated cells were analyzed as a control group. **(B)** RNA was extracted from cells in time points: days 5, 7, 9, 14, 21 and mRNA expression was detected by qRT-PCR. Runx2, OSX, BSP and OC/BGKAP2mRNA expression normalized to undifferentiated cells control group. **(C)** In 10, 14 and 21 days, cells were blocked in paraformaldehyde 4% and stained for Alizarin red staining (above), and numerical evaluation for Alizarin red optical density in 45 nm was calculated (graph below) (days 10, 14 21, N=5-6/group). The asterisk symbol represents a statistical significance *<0.05; **< 0.01; ***< 0.005.

MC3T3-E1 preosteoblasts were cultured with or without RvD1 200 nM for 21 days (**Figure 5A**). RvD1 increased osteoblastogenesis markers during time: mRNA expression of Runx2 and BSP were observed at a higher level in the RvD1 treatment group on day 7. In most of the time points (days 5, 9, 14, 21) RvD1 treatment upregulated OSX expression compared to the control of the differentiating cell. OC/Bglap2 (osteocalcin/bone gamma-carboxyglutamate (gla) protein 2) exhibited the same trend until day 14. Later OC/Bglap2 expression was significantly downregulated in comparison to differentiating cells control (**Figure 5B**).

The extracellular calcium deposit was stained *via* Alizarin-red staining for 10, 14 and 21 days. Differentiated cells that were treated with RvD1 exhibited redder plaques in day 10, and darker staining at days 14 and 21, indicating higher accumulative calcium layers in the well. Undifferentiated cells were slightly reddish as a control. Alizarin red optical density in 405 nm numerical values revealed increased calcium staining in RvD1 differentiated cells compared to the differentiated cells control group (**Figure 5C**) (days 10, 14 n=6, day 21 n=5 * *P<*0.05 *** *P<* 0.005).

## Discussion

The results of our study indicate that in addition to control of inflammation and to the anti-catabolic effect, repeated administration of RvD1 has a bone regenerative effect, *via* enhanced osteoblasts differentiation and secretion of anabolic factors.

In the mRNA sequencing analysis, there were no significant pathways or prominent genes specifically related to bone. In contrast, allograft increased the expression of inflammatory markers, IL1RL1 and NR1D1, which might explain the delay in bone healing at this time point. IL1RL1 is coding to ST2 protein, (the receptor of IL-33) which is usually related to TH2 cells (34, 35). The literature provides conflicting evidence regarding ST2’s effect on bone. Some studies showed that ST2 is mediating human degenerative diseases associated with bone and cartilage destruction (36, 37). NR1D1 is overexpressed in osteoarthritis, in which it negatively regulates the expression of OPG and decreases osteoblasts differentiation (26).

We decided to limit the duration of the experiment at 2 weeks post implantation, as we intended to study the effect of RvD1 before bone regeneration. This was based on our previous study, in which we showed that bone healing was impaired in presence of allograft particles at 2 weeks post implantation, whereas at 6 weeks regeneration already occurred (24).

A single administration of RvD1 did not improve bone regeneration and allograft osteointegration, probably because of its relatively short-term effect, as shown by its fast clearance from the site of administration (**Figure 2A**) The kinetics experiment showed that RvD1 was flushed away in less than 24 hours. These results are supportive of previous reports on RvD1 pharmacokinetics (38).

Our working hypothesis was that repeated administration (Rep-RvD1) will result in longer exposure of the bone to RvD1 and therefore it will improve its therapeutic efficacy. To prolong RvD1’s presence near the alveolar bone socket we conducted three sub-gingival injections.

Repeated administration of RvD1 increased BV/TV not only compared with allograft implantation treatment groups, but also compared with Spon. Healing. Additionally, RvD1 increased *de-novo* osteoid apposition.

Previous attempts to improve bone healing with RvD1 were made. Xiaofeng et al. demonstrated improved bone healing in rat-calvaria model. Rats were treated with collagen scaffolds, in addition to weekly subcutaneous (SC) injections of RvD1 (39). Subcutaneous tissue enables slow release of molecules due to its special tissue architecture and its combination with collagen scaffolds, thereby reducing the need for frequent injections and their related side effects. In contrast, in our study RVD1 was injected into attached gingiva, a vascular and sparse tissue which allows rapid dissipation. In addition, we aimed to investigate the RvD1 local effect and therefore we chose to administrate it locally and not SC.

So far, resolvins, in general and RvD1 in particular, were mentioned as beneficials in bone degeneration conditions, due to their anti-inflammatory effect (12–15, 18). Vasconcelos et al. showed that chitosan porous 3D scaffolds embedded with RvD1 improved bone healing (17). They showed that RvD1 preserved bone by a traditional effect of immunomodulation and not by an active anabolic process.

Our results provide further support for these findings, as we show that rep-RvD1 decreases the IL1RL1 and NR1D1 expression, which are both inflammatory indicators that were increased following allograft implantation.

Furthermore, we show for the first time a direct anabolic effect of RvD1 *in vivo* & *in vitro*. Rep-RvD1 increased RUNX2, OSX, BMP-5 and Amlex expression compared to rep-sham*, in-vivo*. Runx2 is a well-known factor of osteoblasts differentiation, bone formation, and mineralization (40). Runx2 is crucial for optimal bone metabolism in many bone conditions and is considered one of the master markers of osteoblastic activity. Increased Runx2 expression *in vivo* is correlative with improved bone parameters, as demonstrated in this research and supported by previous data (40, 41). OSX is also expressed in osteoblast- lineage cells and serves as a transcription factor that induces the expression of collagen type1, osteocalcin (OC) and Bone sialoprotein (BSP) (41, 42). BMP5 inhibits osteoclastogenesis (43) and promotes osteogenesis (44). AMLEX is a growth factor-like molecule expressed in alveolar bone, long bone and cartilage and is associated with enhanced osteogenic differentiation (45).

*In vitro*, repeated administration of RvD1 directly increased osteoblasts differentiation. Known osteoblastogenesis markers (40, 46), indicative of osteoblastic activity were evaluated, as well as the calcified matrix that was secreted from the cells. RvD1 multiplied the expression of RUNX2 by almost 4 and doubled the expression of BSP on day 7. OSX and OC/Bglap were also significantly increased in most of the time points, except for a decrease in OC/Bglap2 on day 21. Differentiated cells that were treated with RvD1 demonstrated higher mineralized extracellular matrix, and higher optical density compared with differentiated osteoblasts without RvD1. These results are in contrast to Coetzee et al., which showed that arachidonic acid and docosahexaenoic acid did not enhance MCT3E1 cell differentiation into osteoblasts after 48 hours (47). However, in our experiment we tested the MCT3E1 cell differentiation for a longer period, by utilizing the continuous effect of repeated administration of RvD1.

Our results support the well-known RvD1 anti-catabolic effect, since Rep-RvD1 decreased the osteoclast’ number compared to all allograft controls. The TRAP analysis revealed that osteoclasts numbers increased in all allograft groups compared with Spon. Healing, supporting previous study in dogs, which showed that when tooth extraction sockets were filled with Bio-oss collagen grafts, osteoclasts were involved in graft incorporation (48).

Moreover, RvD1 had a significant effect on RANKL/OPG ratio *in vitro*, a pivotal pathway for osteoclastogenesis induction. IL-17 inflammatory environment increased RANKL/OPG ratio, while the presence of RvD1 significantly reduced this ratio, thus decreasing osteoclastic activity. These findings are in concordance to previous studies on RvE1. Gao et al. showed that RvE1 increased the balance of OPG secretion levels from osteoblasts in IL-6 inflammatory conditions (18), while Funaki et al. reported a decreased RANKL expression in osteoblasts cultured with Il-17, without a change in OPG expression (49).

**Figure 6:**
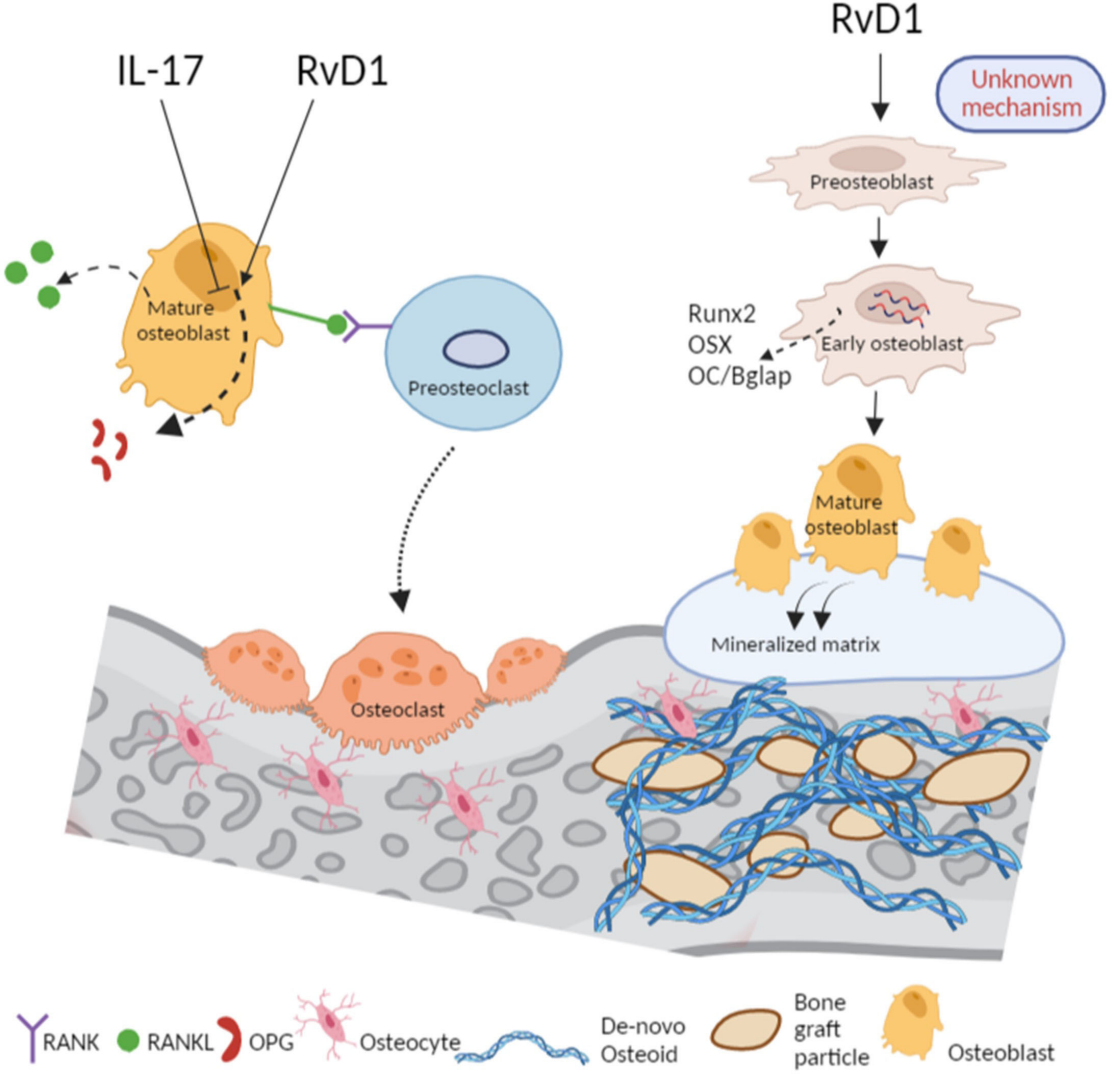
RvD1 promotes bone healing *via* inhibition of osteoclastogenesis and accelerating osteoblastogenesis. RvD1 controls bone healing *via* dual mechanisms: From left, in proinflammatory conditions of IL-17, the RANKL/OPG ratio is increased following a decrease of OPG thus, promoting osteoclastogenesis. In contrast, and in spite of the presence of IL-17 in the medium, RvD1 increases OPG secretion from osteoblasts, thus, leading to reduction of RANKL/OPG ratio and osteoclastogenesis inhibition. From right, RvD1 as an adjuvant in the medium of vitro cell culture of preosteoblasts directly effects them to express higher osteogenesis markers such as Runx2, OSX and OC/Bglap. Therefore, RvD1 accelerates the development of osteoblasts and their secretion of calcified matrix. *In vivo*, RvD1 enhances the apposition of osteoid and improves bone regeneration. The figure was created with Biorender.

The dual role of RvD1 in bone regeneration is summarized in a scheme (**Figure 6**). Firstly, RvD1 indirectly reduces osteoclastogenesis by elevating the OPG secretion from pre-osteoblasts without altering RANKL secretion, thereby decreasing RANKL/OPG ratio. Secondly, RvD1 has an anabolic effect as it increases osteoblastogenesis *in vitro* and allograft osteointegration and new bone formation, *in vivo*.

To the best of our knowledge, we are the first to demonstrate that RvD1 positively and directly affects osteoblast function, as manifested through increased expression of osteoblasts key markers of differentiation and through enhanced secretion of bone matrix. Although future experiments are needed to further unravel its mechanism of action on osteoblasts, this novel result is of significance to the osteoimmunology field and expands the potential clinical uses of RvD1.

Despite these encouraging results, the use of the repetitive administration has several drawbacks. Firstly, it impaired bone healing by almost 30% when compared to single sham treatment (**Supplementary Figure A**), due to repeated injury to the healing tissues (50). Subsequently, although rep-RvD1 improved bone healing by~130% compared with rep-sham, it only increased the BV/TV ratio by 36% compared with Spon. Healing control (**Figure 2G**). Secondly, repeated administration requires repetitive anesthesia, which weakened the mice and led to weight loss (**Supplementary Figure 2F**) (51). We assume that repeated anesthesia decreased motor activity and food consumption (52) and inflicted intra-oral pain. Thirdly, repetitive administrations are also not clinically viable (53, 54).

Despite these limitations, our study demonstrates that prolonged exposure of bone tissue to RvD1 has an anabolic effect and enhances bone regeneration *via* a dual mechanism: inhibition of osteoclasts and promotion of differentiation and functionality of osteoblasts.

## Conclusion

1. A single administration of RvD1 as-is has no therapeutic effect due to its rapid clearance from the administration site.
2. Prolonged exposure of bone to RvD1 can overcome this limitation, but is associated with side effects.
3. Repeated RvD1 administration has a bone regenerative effect *via* a dual mechanism: suppression of osteoclastogenesis and enhanced osteoblasts differentiation and functionality and secretion of anabolic factors.

RvD1 bioagent possesses promising features that justify further research on its potential integration in various bone related fields such as implantology, degenerative diseases and treatment of non-union fractures. Due to the downsides of repeated injections, future studies should aim to develop an RvD1 sustained release delivery system which will reduce treatment frequency and the associated damage, while preserving the clinical therapeutic effectiveness.

## Data availability statement

The datasets presented in this study can be found in online repositories. The names of the repository/repositories and accession number(s) can be found below: GSE224195 (GEO).

## Ethics statement

The animal study was reviewed and approved by Animal Care and Use Committee of the Hebrew University.

## Author contributions

NP- Contributed to the conception, study design, data acquisition, interpretation, drafted and critically revised the manuscript. YK- Contributed to the conception, study design, interpretation, and critically revised the manuscript. ED- Contributed to the conception, interpretation and critically revised the manuscript. DP- Contributed to the conception, interpretation and critically revised the manuscript. DS- Contributed to the conception and study design. YKH Contributed to the conception and study design and critically revised the manuscript. GM- Contributed to the conception and study design. YB- Contributed to the conception study design, interpretation and critically revised the manuscript. SC- Contributed to the conception study design, interpretation and critically revised the manuscript. All authors contributed to the article and approved the submitted version.

## Supplementary material

Supplementary Figure 1
**(A)** Weight increase (% from the initial weight) in days 4, 7, and 14 post ABR Figure

Supplementary Figure 2
**(A)** Bone volume/total volume (BV/TV) of rep-sham administration normalized to single sham administration. **(B)** Weight increase (% from the initial weight) in days 4, 7, 8 and 14 post-ABR of all groups.

## References

[1] Fesseha H Fesseha Y. Bone grafting, its principle and application: A review. Osteol Rheumatol Open J (2020) 1(1):43–50. doi: 10.17140/ORHOJ-1-113

[2] Peres JA Lamano T. Strategies for stimulation of new bone formation: A critical review. Braz Dental J (2011) 22(6). doi: 10.1590/S0103-64402011000600001 22189637

[3] Zhang M Matinlinna JP Tsoi JKH Liu W Cui X Lu WW. Recent developments in biomaterials for long-bone segmental defect reconstruction: A narrative overview. J Orthopaedic Transl (2020) 22:26–33. doi: 10.1016/j.jot.2019.09.005 PMC7231954 32440496

[4] Kowalczewski CJ Saul JM. Biomaterials for the delivery of growth factors and other therapeutic agents in tissue engineering approaches to bone regeneration. Front Pharmacol (2018) 9. doi: 10.3389/fphar.2018.00513 PMC5986909 29896102

[5] Phan TCA Xu J Zheng MH. Interaction between osteoblast and osteoclast: Impact in bone disease. Histol Histopathol (2004) 19:4. doi: 10.14670/HH-19.1325 15375775

[6] Krishnakumar GS Roffi A Reale D Kon E Filardo G. Clinical application of bone morphogenetic proteins for bone healing: a systematic review. Int Orthopaedics (2017) 41(6):1073–83. doi: 10.1007/s00264-017-3471-9 28424852

[7] Skovrlj B Koehler SM Anderson PA Qureshi SA Hecht AC Iatridis JC. Association between BMP-2 and carcinogenicity. Spine (2015) 40(23):1862–71. doi: 10.1097/BRS.0000000000001126 PMC7423158 26274524

[8] Bach DH Park HJ Lee SK. The dual role of bone morphogenetic proteins in cancer. Mol Ther - Oncol (2018) 8:1–13. doi: 10.1016/j.omto.2017.10.002 PMC5723373 29234727

[9] Baht GS Vi L Alman BA. The role of the immune cells in fracture healing. Curr Osteoporosis Rep (2018) 16(2):138–45. doi: 10.1007/s11914-018-0423-2 PMC5866272 29508143

[10] Thomas MV Puleo DA. Infection, inflammation, and bone regeneration: A paradoxical relationship. J Dental Res (2011) 90(9):1052–61. doi: 10.1177/0022034510393967 PMC3169879 21248364

[11] Kain V Ingle KA Colas RA Dalli J Prabhu SD Serhan CN. Resolvin D1 activates the inflammation resolving response at splenic and ventricular site following myocardial infarction leading to improved ventricular function. J Mol Cell Cardiol (2015) 84:24–35. doi: 10.1096/fasebj.29.1_supplement.285.4 PMC4468047 25870158

[12] Benabdoune H Rondon EP Shi Q Fernandes J Ranger P Fahmi H. The role of resolvin D1 in the regulation of inflammatory and catabolic mediators in osteoarthritis. Inflam Res (2016) 65(8):635–45. doi: 10.1007/s00011-016-0946-x 27056390

[13] Murakami K. Potential of specialized pro-resolving lipid mediators against rheumatic diseases. Japanese J Clin Immunol (2016) 39(3):155–63. doi: 10.2177/jsci.39.155 27320930

[14] Norling LV Headland SE Dalli J Arnardottir HH Haworth O Jones HR. Proresolving and cartilage-protective actions of resolvin D1 in inflammatory arthritis. JCI Insight (2016) 1(5). doi: 10.1172/jci.insight.85922 PMC4855303 27158677

[15] Benabdoun HA Kulbay M Rondon EP Vallières F Shi Q Fernandes J. *In vitro* and *in vivo* assessment of the proresolutive and antiresorptive actions of resolvin D1: Relevance to arthritis. Arthritis Res Ther (2019) 21(1):72. doi: 10.1186/s13075-019-1852-8 30867044 PMC6416871

[16] Klein Y Shani-Kdoshim S Maimon A Fleissig O Levin-Talmor O Meirow Y. Bovine bone promotes osseous protection *via* osteoclast activation. J Dent Res (2020) 99(7):820–9. doi: 10.1177/0022034520911647 32167856

[17] Vasconcelos DP Costa M Neves N Teixeira JH Vasconcelos DM Santos SG. Chitosan porous 3D scaffolds embedded with resolvin D1 to improve in vivo bone healing. J BioMed Mater Res - Part A. (2018) 106(6):1626–33. doi: 10.1002/jbm.a.36370 29453815

[18] Gao L Faibish D Fredman G Herrera BS Chiang N Serhan CN. Resolvin E1 and chemokine-like receptor 1 mediate bone preservation. J Immunol (2013) 190(2):689–94. doi: 10.4049/jimmunol.1103688 PMC3538964 23241890

[19] Steffens JP Herrera BS Coimbra LS Stephens DN Rossa C Spolidorio LC. Testosterone regulates bone response to inflammation. Horm Metab Res (2014) 46(3):193–200. doi: 10.1055/s-0034-1367031 PMC4522923 24526374

[20] Natto ZS Parashis A Steffensen B Ganguly R Finkelman MD Jeong YN. Efficacy of collagen matrix seal and collagen sponge on ridge preservation in combination with bone allograft: A randomized controlled clinical trial. J Clin Periodontol (2017) 44(6):649–59. doi: 10.1111/jcpe.12722 28303642

[21] Marttila E Grönholm L Saloniemi M Rautemaa-Richardson R. Prevalence of bacteraemia following dental extraction–efficacy of the prophylactic use of amoxicillin and clindamycin. Acta Odontol Scand (2021) 79(1):25–30. doi: 10.1080/00016357.2020.1768285 32449864

[22] Steiner GG Francis W Burrell R Kallet MP Steiner DM Macias R. The healing socket and socket regeneration. Compend Contin Educ Dent (2008) 29(2):114–6. 18429427

[23] Klein Y Levin-talmor O Berkstein JG Wald S Meirow Y Maimon A. Resolvin D1 shows osseous- protection *via* RANK reduction on monocytes during orthodontic tooth movement. Front Immunol. (2022) 13:1–14. doi: 10.3389/fimmu.2022.928132 PMC9585452 36275768

[24] Klein Y Kunthawong N Fleissig O Casap N Polak D Chaushu S. The impact of alloplast and allograft on bone homeostasis: Orthodontic tooth movement into regenerated bone. J Periodontol (2020) 91(8):1067–75. doi: 10.1002/JPER.19-0145 31887228

[25] Klein Y Fleissig O Stabholz A Chaushu S Polak D. Bone regeneration with bovine bone impairs orthodontic tooth movement despite proper osseous wound healing in a novel mouse model. J Periodontol (2019). doi: 10.1002/JPER.17-0550 30059146

[26] Hirai T Tanaka K Togari A. α1B -adrenergic receptor signaling controls circadian expression of Tnfrsf11b by regulating clock genes in osteoblasts. Biol Open (2015) 4(11):1400–9. doi: 10.1242/bio.012617 PMC4728343 26453621

[27] Bouxsein ML Boyd SK Christiansen BA Guldberg RE Jepsen KJ Müller R. Guidelines for assessment of bone microstructure in rodents using micro-computed tomography. J Bone Miner Res (2010) 25(7):1468–86. doi: 10.1002/jbmr.141 20533309

[28] Kuroshima S Kovacic BL Kozloff KM McCauley LK Yamashita J. Intra-oral PTH administration promotes tooth extraction socket healing. J Dent Res (2013). doi: 10.1177/0022034513487558 PMC3654759 23611925

[29] Klein Y Fleissig O Polak D Barenholz Y Mandelboim O Chaushu S. Immunorthodontics: *In vivo* gene expression of orthodontic tooth movement. Sci Rep (2020) 10(1):8172. doi: 10.1038/s41598-020-65089-8 32424121 PMC7235241

[30] Hashimshony T Senderovich N Avital G Klochendler A de Leeuw Y Anavy L. CEL-Seq2: sensitive highly-multiplexed single-cell RNA-seq. Genome Biol (2016) 17(1):1–7. doi: 10.1186/s13059-016-0938-8 27121950 PMC4848782

[31] Feng Y Su L Zhong X Wei G Xiao H Li Y. Exendin-4 promotes proliferation and differentiation of MC3T3-E1 osteoblasts by MAPKs activation. J Mol Endocrinol (2016). doi: 10.1530/JME-15-0264 26647389

[32] Bayraktar S Jungbluth P Deenen R Grassmann J Schneppendahl J Eschbach D. Molecular- and microarray-based analysis of diversity among resting and osteogenically induced porcine mesenchymal stromal cells of several tissue origin. J Tissue Eng Regener Med (2018). doi: 10.1002/term.2375 PMC5811815 27966263

[33] Glueck M Gardner O Czekanska E Alini M Stoddart MJ Salzmann GM. Induction of osteogenic differentiation in human mesenchymal stem cells by crosstalk with osteoblasts. Biores Open Access (2015) 4(1):121–30. doi: 10.1089/biores.2015.0002 PMC4497645 26309789

[34] Griesenauer B Paczesny S. The ST2/IL-33 axis in immune cells during inflammatory diseases. Front Immunol (2017) 8:475. doi: 10.3389/fimmu.2017.00475 28484466 PMC5402045

[35] Li J Tan J Martino MM Lui KO. Regulatory T-cells: Potential regulator of tissue repair and regeneration. Front Immunol (2018) 9. doi: 10.3389/fimmu.2018.00585 PMC5890151 29662491

[36] Murphy GEJ Xu D Liew FY McInnes IB. Role of interleukin 33 in human immunopathology. Ann Rheum Dis (2010) 69. doi: 10.1136/ard.2009.120113 19995743

[37] Xu D Jiang HR Kewin P Li Y Mu R Fraser AR. IL-33 exacerbates antigen-induced arthritis by activating mast cells. Proc Natl Acad Sci USA (2008) 105(31). doi: 10.1073/pnas.0801898105 PMC2491487 18667700

[38] Yellepeddi VK Parashar K Dean SM Watt KM Constance JE Baker OJ. Predicting resolvin D1 pharmacokinetics in humans with physiologically-based pharmacokinetic modeling. Clin Transl Sci (2021) 14(2):683–91. doi: 10.1111/cts.12930 PMC7993257 33202089

[39] Jiang X Liu J Li S Qiu Y Wang X He X. The effect of resolvin D1 on bone regeneration in a rat calvarial defect model. J Tissue Eng Regener Med (2022) 16(11):987–97. doi: 10.1002/term.3345 PMC9804777 35980287

[40] Wu H Whitfield TW Gordon JAR Dobson JR Tai PWL van Wijnen AJ. Genomic occupancy of Runx2 with global expression profiling identifies a novel dimension to control of osteoblastogenesis. Genome Biol (2014) 15(3):1–17. doi: 10.1186/gb-2014-15-3-r52 PMC4056528 24655370

[41] Liu Q Li M Wang S Xiao Z Xiong Y Wang G. Recent advances of osterix transcription factor in osteoblast differentiation and bone formation. Front Cell Dev Biol (2020) 8:601224. doi: 10.3389/fcell.2020.601224 33384998 PMC7769847

[42] Rath B Nam J Knobloch TJ Lannutti JJ Agarwal S. Compressive forces induce osteogenic gene expression in calvarial osteoblasts. J Biomech (2008) 41(5):587–92. doi: 10.1016/j.jbiomech.2007.11.024 PMC2291547 18191137

[43] Lademann F Hofbauer LC Rauner M. The bone morphogenetic protein pathway: The osteoclastic perspective. Front Cell Dev Biol (2020) 8(October). doi: 10.3389/fcell.2020.586031 PMC7597383 33178699

[44] Yazawa M Kishi K Nakajima H Nakajima T. Expression of bone morphogenetic proteins during mandibular distraction osteogenesis in rabbits. J Oral Maxillofac Surg (2003). doi: 10.1053/joms.2003.50116 12730838

[45] Wang F Okawa H Kamano Y Niibe K Kayashima H Osathanon T. Controlled osteogenic differentiation of mouse mesenchymal stem cells by tetracycline-controlled transcriptional activation of amelogenin. PloS One (2015). doi: 10.1371/journal.pone.0145677 PMC4692545 26709694

[46] Fu C Yang X Tan S Song L. Enhancing cell proliferation and osteogenic differentiation of MC3T3-E1 pre-osteoblasts by BMP-2 delivery in graphene oxide-incorporated PLGA/HA biodegradable microcarriers. Sci Rep (2017) 7(1):12549.2. doi: 10.1038/s41598-017-12935-x 28970533 PMC5624967

[47] Coetzee M Haag M Kruger MC. Effects of arachidonic acid and docosahexaenoic acid on differentiation and mineralization of MC3T3-E1 osteoblast-like cells. Cell Biochem Funct (2009) 27:3–11. doi: 10.1002/cbf.1526 19107879

[48] Araújo MG Liljenberg B Lindhe J. Dynamics of bio-oss® collagen incorporation in fresh extraction wounds: An experimental study in the dog. Clin Oral Implants Res (2010) 21(1):55–64. doi: 10.1111/j.1600-0501.2009.01854.x 20070748

[49] Funaki Y Hasegawa Y Okazaki R Yamasaki A Sueda Y Yamamoto A. Resolvin E1 inhibits osteoclastogenesis and bone resorption by suppressing IL-17-induced RANKL expression in osteoblasts and RANKL-induced osteoclast differentiation. Yonago Acta Med (2018) 61(1):8–18. doi: 10.33160/yam.2018.03.002 PMC5871721 29599617

[50] Eming SA Martin P Tomic-Canic M. Wound repair and regeneration: Mechanisms, signaling, and translation. Sci Trans Med (2014) 6:265. doi: 10.1126/scitranslmed.3009337 PMC4973620 25473038

[51] Dholakia U Clark-Price SC Keating SCJ Stern AW. Anesthetic effects and body weight changes associated with ketamine-xylazine-lidocaine administered to CD-1 mice. PloS One (2017) 12(9). doi: 10.1371/journal.pone.0184911 PMC5599034 28910423

[52] Bajwa NM Lee JB Halavi S Hartman RE Obenaus A. Repeated isoflurane in adult male mice leads to acute and persistent motor decrements with long-term modifications in corpus callosum microstructural integrity. J Neurosci Res (2019) 97(3):332–45. doi: 10.1002/jnr.24343 30394562

[53] Saperov VN. Cooperation with patients - guarantee of successful treatment. Clin Med (Russ J) (2020) 94(7). doi: 10.18821/0023-2149-2016-94-7-554-559 30289223

[54] Walters-Salas T. The challenge of patient adherence. Bariatric Nurs Surg Patient Care (2012) 17. doi: 10.1089/bar.2012.9960

