# Supplementary material for: Resolvin D1 improves allograft osteointegration and directly enhances osteoblasts differentiation
Source: Front Immunol. 2023 Feb 27;14:1086930. doi: 10.3389/fimmu.2023.1086930 (PMC10008843; doi:10.3389/fimmu.2023.1086930)
Supplement: Supplementary file 1 [file Table_1.pdf]

Gene	Primer Sequence 5' → 3'
NR1D1	
Forward	ACGACCCTGGACTCCAATAA
Reverse	CCATTGGAGCTGTCACTGTAGA
IL1RL1	
Forward	ACGCTCGACTTATCCTGTGG
Reverse	CAGGTCAATTGTTGGACACG
OPG	
Forward	TGGAACCCCAGAGCGAAACA
Reverse	GCAGGAGCCAAATGTGCTG
RANKL	
Forward	TGTACTTTCGAGCGCAGATG
Reverse	AGGCTTGTTTCATCCTCCTG
Runx2 a	
Forward	TCCACCACGCCGCTGTCT
Reverse	TCAGTGAGGGATGAAATGCT
OSX	
Forward	CCCTTCTCAAGCACCAATGG
Reverse	AGGGTGGGTAGTCATTTGCATAG
OC/BGLP2	
Forward	CTGACAAAGCCTTCATGTCCAA
Reverse	GCGCCGGAGTCTGTTCACTA
GAPDH	
Forward	TTGATGGCAACAATCTCCAC
Reverse	CGTCCCGTAGACAAAATGGT
