# Supplementary figures and images for: Resolvin D1 improves allograft osteointegration and directly enhances osteoblasts differentiation
Source: Front Immunol. 2023 Feb 27;14:1086930. doi: 10.3389/fimmu.2023.1086930 (PMC10008843; doi:10.3389/fimmu.2023.1086930)

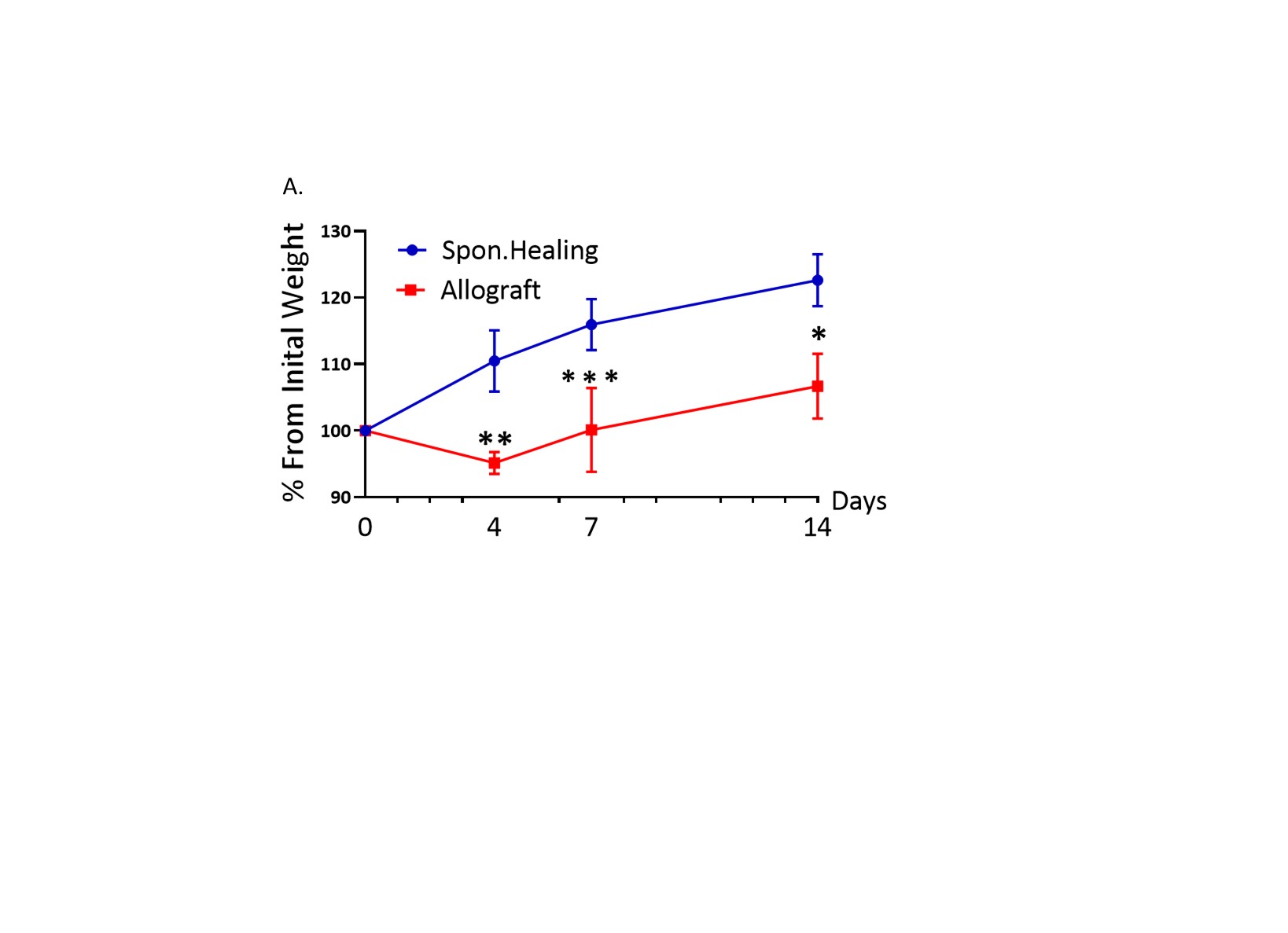

Supplement: Supplementary Figure 1 — (A) Weight increase (% from the initial weight) in days 4, 7, and 14 post ABR Figure [file Image_1.jpeg]

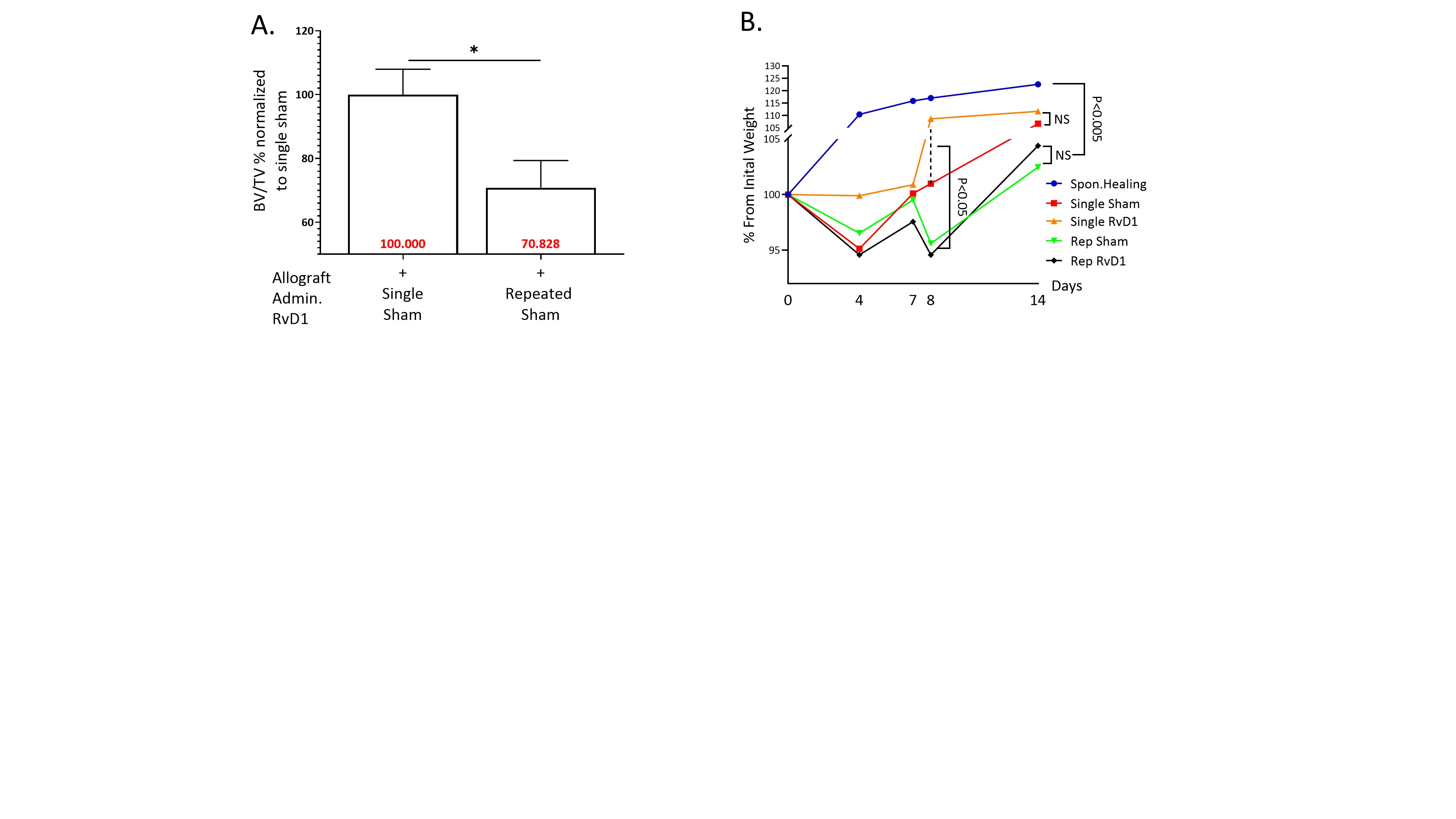

Supplement: Supplementary Figure 2 — (A) Bone volume/total volume (BV/TV) of rep-sham administration normalized to single sham administration. (B) Weight increase (% from the initial weight) in days 4, 7, 8 and 14 post-ABR of all groups. [file Image_2.jpeg]
